# Supplementary material for: Causal interplay between lactose intolerance and gut microbiota: a combined bidirectional Mendelian randomization and in vivo validation study
Source: Front Nutr. 2026 Jun 1;13:1803337. doi: 10.3389/fnut.2026.1803337 (PMC13265576; doi:10.3389/fnut.2026.1803337)
Supplement: Supplementary file 1 [file Data_Sheet_1.zip › supplementary materials/Forward/leave one out cross validation/ebi-a-GCST90027474.finngen_R12_E4_LACTONAS.pdf]

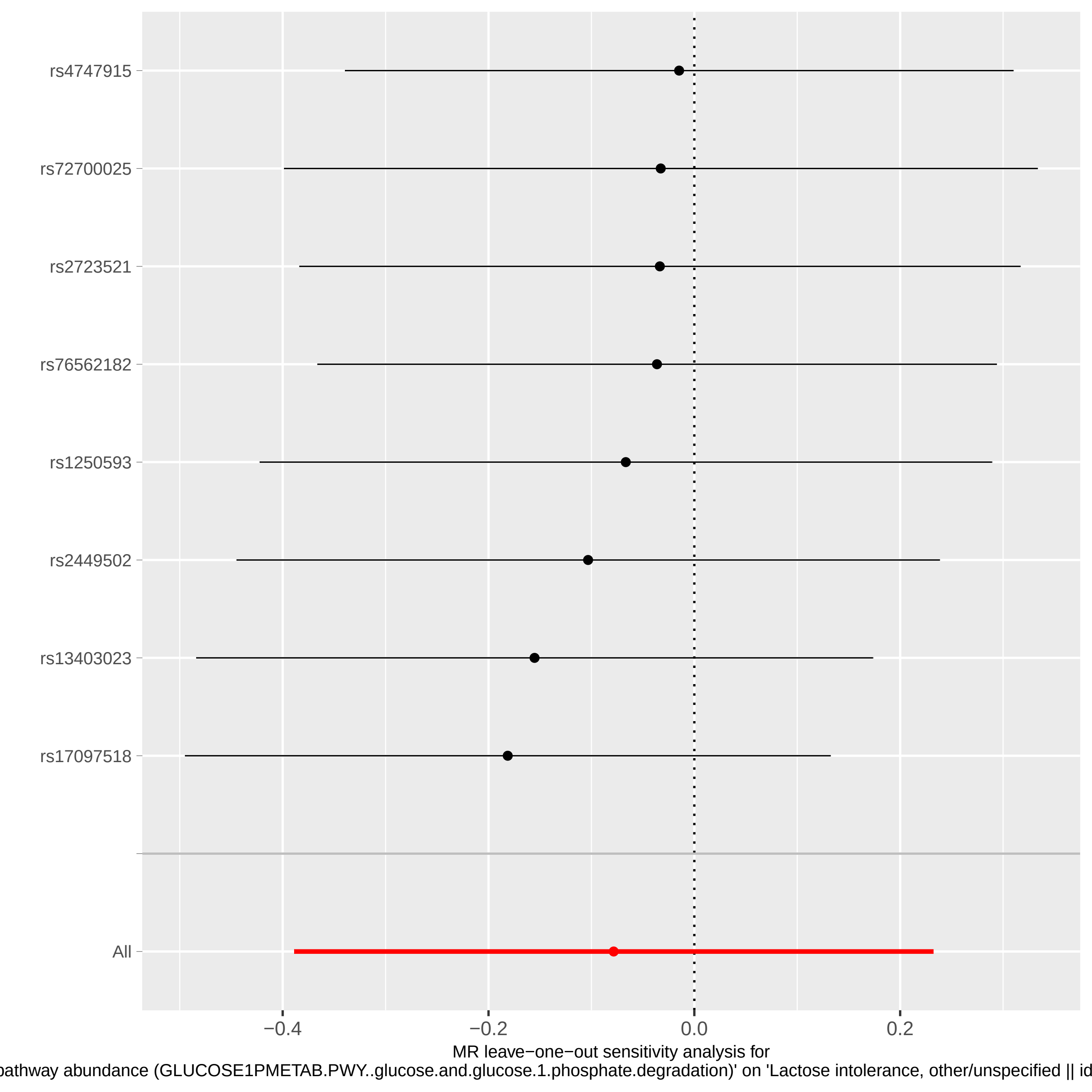

rs4747915
rs72700025
rs2723521
rs76562182
rs1250593
rs2449502
rs13403023
rs17097518
All
−0.4
−0.2
0.0
0.2
MR leave−one−out sensitivity analysis for
pathway abundance (GLUCOSE1PMETAB.PWY..glucose.and.glucose.1.phosphate.degradation)' on 'Lactose intolerance, other/unspecified || id
